# SO _2_ sorption properties of fly ash zeolites

**DOI:** 10.3906/kim-1905-50

**Published:** 2020-02-11

**Authors:** Natalia CZUMA, Wojciech FRANUS, Paweł BARAN, ĆWIK Agnieszka, Katarzyna ZARĘBSKA

**Affiliations:** 1 Department of Coal Chemistry and Environmental Sciences, Faculty of Energy and Fuels, AGH University of Science and Technology, Kraków Poland; 2 Department of Geotechnical Science, Faculty of Civil Engineering and Architecture, Lublin University of Technology, Lublin Poland; 3 Institute of Energy Technologies, Polytechnic University of Catalonia (UPC), Barcelona Spain; 4 Barcelona Research Center in Multiscale Science and Engineering, Barcelona Spain

**Keywords:** SO _2_ sorption, fly ash, zeolite, water vapor

## Abstract

In the presented study, the sulfur dioxide sorption properties of fly ash zeolite X were investigated. Sorption tests were performed on fly ash zeolite samples that were not prepared specially for sorption, in addition to dried samples and samples in the presence of water vapor. The samples saturated with water vapor showed the highest sorption capacity. The sorption capacity of the samples additionally dried prior to the sorption experiment was higher than that of the samples that were not specially prepared for the sorption test. Regeneration tests indicated relatively good regeneration properties. The obtained results were described with the use of Langmuir, Sips, and Dubinin–Astakhov models, with the Dubinin–Astakhov model providing the best fit.

## 1. Introduction

Fly ash comprises byproduct materials remaining from the process of fossil-fuel combustion. Fly ash formed in the process of powdered coal combustion leaves the furnace (boiler) with the exhaust gas stream and is captured using electrostatic precipitators (ESPs). It is characterized by its fine mineral dust form [1]. According to the source (fuel type, the type of furnace, use of biomass addition, and desulfurization process), fly ash has different chemical compositions [2]. The main components include SiO _2_ , Al _2_ O _3_ , Fe _2_ O _3_ , and CaO. The composition is complemented by secondary components, e.g., MgO, SO , Na _2_ O, K _2_ O, and trace components such as TiO _2_ , P _2_ O _5_ , MnO, etc. Unburned carbon fragments may also be found in fly ash. 1Kasprzyk K, Pietrykowski P. Wykorzystanie popiołów lotnych w gospodarce [online]. Website http://www.spalanie.pwr.wroc.pl/badania/witryfikacja/popioly.htm [accessed 27 June 2019].
Despite the fact that fly ash has different chemical compositions, the main compositions mean that fly ash is an economically and ecologically attractive waste material for reuse. Fly ash can be used in many areas, such as in the production of construction materials and cement, or in the road-building industry and mining. One possible fly ash usage is as a substrate in the process of zeolite synthesis [2–6].

Zeolites are crystalline aluminosilicates containing pores and channels of molecular dimensions and they have a wide spectrum of applications [5–7]. Their properties allow the use of these materials in the purification of gases, sewage, and groundwater and in soil remediation, etc. [4,8–11]. The molecular sieving properties of fly ash zeolites confirm their potential use as a SO _2_ sorbent for flue gases [12–14].

For many years, extensive research has been undertaken to investigate possible approaches to minimize SO _2_ emissions from industrial sources. Generally, the techniques used include regenerative and nonregenerative methods. Both methods are characterized by some advantages and limitations. In the case of nonregenerative methods, the limitation is the need for natural resources with strictly determined purity parameters, and there are increased energy penalties in the case of regenerative methods [15]. Therefore, research based on the development of new sorbents, which would have a positive influence on the energy balance of the process of capturing of SO _2_ , is fully justified.

There is a lack of scientific studies that combine the efficiency of SO _2_ capture with the sorbent preparation process. The sorbent preparation procedure has a direct effect on its sorption properties.

The relatively small amount of research on the influence of the addition/presence of water vapor on the SO _2_ sorption capacity of zeolites provides an incentive to undertake experimental research in this area. In this study, tests were conducted using the hydrothermally synthesized zeolites from fly ash zeolite X.

## 2. Experimental design

The fly ash used was categorized as class F according to American standard ASTM C618-12a. According to this standard, fly ash can be classified as F if it fulfills the following conditions: SiO _2_ + Al _2_ O _3_ + Fe _2_ O ≥ 70% wt., SO ≤ 5% wt., loss on ignition value (LOI) determined at 1273 K, for 1 h ≤ 6% wt., Na _2_ ≤ 1.5% wt. The fly ash was derived from a Polish heat and power plant fired with hard coal and equipped with a pulverized coal-fired boiler.

**Figure 1:**
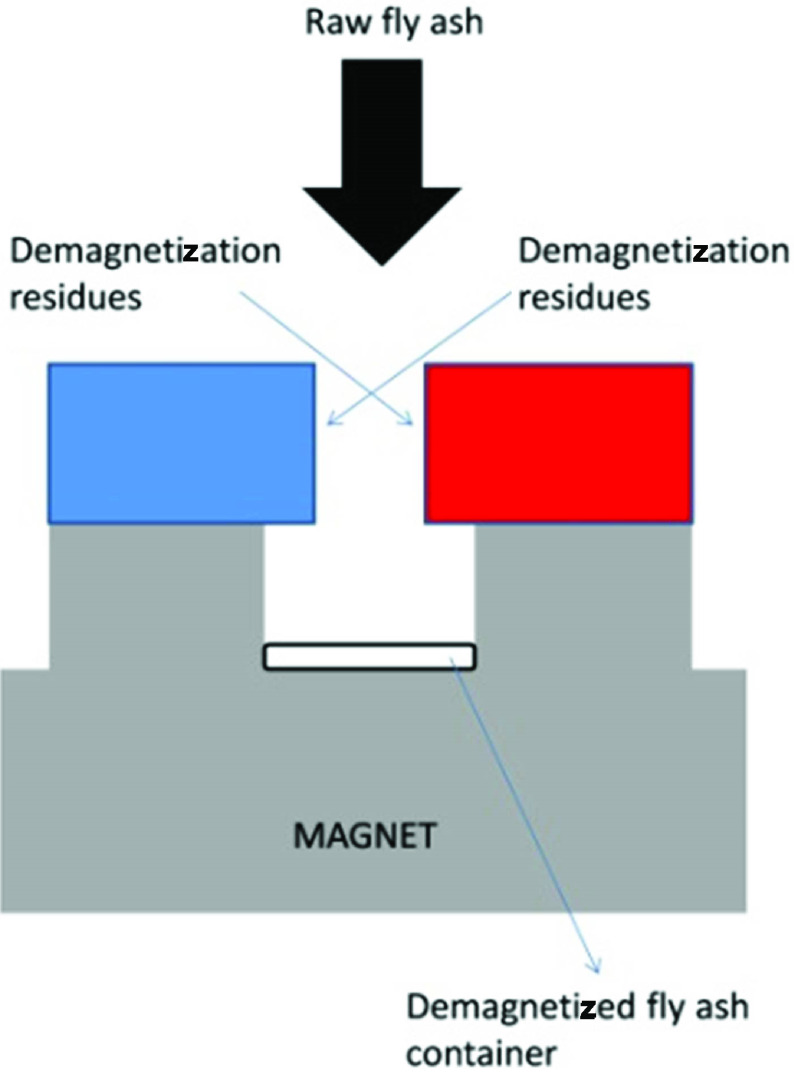
Demagnetization process scheme.

For the experiment, modified fly ash marked with the symbol LO was used. It was generated as a result of the modification of raw fly ash by a magnetic separation process. The process of magnetic separation aimed to eliminate the ferromagnetic materials present in the fly ash. These substances form ballast, which negatively influences the formation of zeolites. The process was performed using a magnet. A schematic representation of the process is presented in Figure 1.

Zeolites synthesized for the process of SO _2_ removal were obtained via hydrothermal fly ash synthesis with an aqueous solution of sodium hydroxide. Hydrothermal synthesis of fly ash zeolites is recognized as a process of fly ash conversion in the presence of an alkaline solution into zeolites. In the hydrothermal synthesis, in the presence of OH _-_ , the Si and Al components are dissolved from the fly ash. The tetrahedra of AlO _5_ and SiO _4_ are the basic building blocks for the zeolite structure. This crystal structure is formed in the presence of Na ^+^ cations. The process of crystallization primarily proceeds on the undissolved or partially dissolved fly ash particles. The incipient crystallization of incompletely dissolved fly ash particles limits the ability of OH _-_ ions to approach the fly ash surface. Accordingly, the fly ash will not dissolve completely and will be transferred to the product. The resulting product consists of unreacted fly ash and zeolite crystals [16].

For these experiments, a solution of 2 mol/dm ^3^ NaOH was mixed with fly ash and with 15% wt. 3 mol/dm ^3^ NaCl. The reaction conditions were a temperature of 373 K and atmospheric pressure. The mixture was protected against excessive evaporation and heated along with mixing for 24 h in the furnace at the given temperature. The obtained material was washed with distilled water several times until the pH was about 10. In the following step, the samples were dried to a constant mass at a temperature of 105 ◦ C.

The elemental composition of the examined fly ash was determined using an Epsilon 3 energy-dispersive X-ray fluorescence spectrometer (PANalytical). Examination of the fly ash was performed in the element range of Na–Am on an apparatus equipped with an RTG Rh 9-W lamp; a 50-kV, 1-mA, 4096 spectrum analyzer; six measuring filters (Cu-500, Cu-300, Ti, Al-50, Al-200, and Ag); and a high-resolution semiconductor SDD detector (Be window of 50-μm thickness), cooled using a Peltier module.

The morphologies and chemical compositions of the microregions of the grains of the main components of the fly ash used for transformation into zeolite were determined using a Quanta 250 FEG scanning electron microscope (SEM) from FEI, with the potential to perform chemical composition analysis by energy-dispersive X-ray microanalysis (EDS).

Roentgen-phase analysis (XRD) was determined via the powder X-ray diffraction method using a PANalytical X’pertPRO MPD with a PW 3020 goniometer. A copper lamp (CuKα = 1.54178 Å) was used as a roentgen radiation source. PANalytical X’Pert HighScore software was used to process the diffraction data. The identification of mineral phases was based on the PDF-2 database (2010 release) formalized by the ICDD.

**Figure 2:**
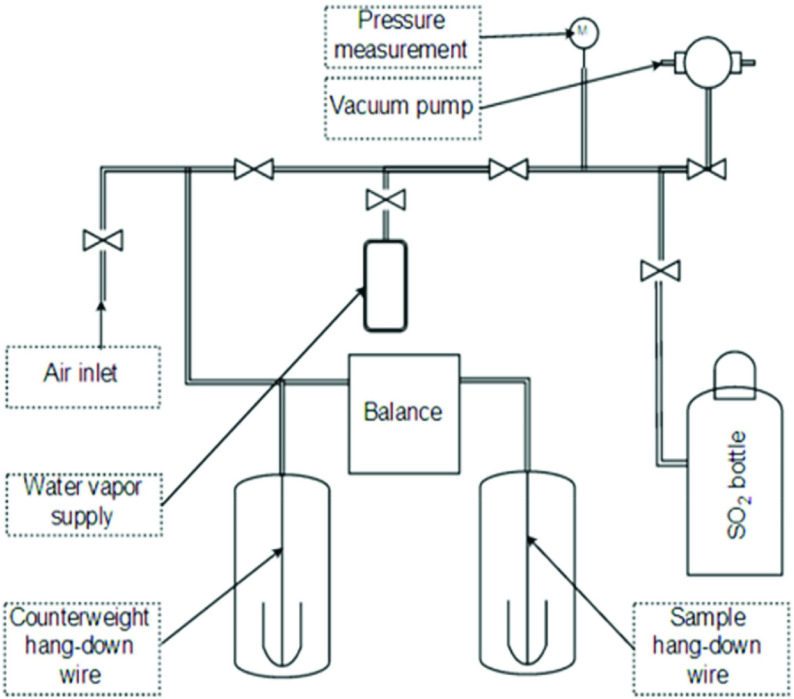
Scheme for SO _2_ sorption experiments.

The obtained zeolite materials were examined with regard to their SO _2_ sorption properties. The research was performed using a Sartorius microbalance, model Electro, with a sensitivity of 0.00005 g and precision of 0.0002 g, presented in Figure 2. The experiment was based on the measurement of the mass increase of the sample once the SO _2_ pressure began to increase. The pressure increase measurement was conducted in time intervals, allowing for the saturation of the samples in each step. The apparatus allowed water vapor to be supplied during the experiment. An air inlet was used for the gas filling of the apparatus once under vacuum. Only sorption curves were recorded. The sorption experiments were performed at room temperature.

Three types of experiments were performed to analyze the influence of the sample preparation and the impact of the presence of water vapor on the SO _2_ sorption capacities of the examined materials:

1. Zeolite material samples were subjected to preliminary drying after the process of synthesis and were kept in zippered sample bags; samples were marked with the letters ZX-WS.

2. In addition to preliminary postsynthesis drying, zeolite material samples were subjected to additional drying under vacuum (1 Pa) at 200 ◦ C and kept in a desiccator; samples were marked with the letters ZX-DS.

3. Zeolite material samples subjected to preliminary drying after synthesis (ZX-WS samples) were tested for the sorption of SO _2_ using a process in which water vapor was present at the initial stage of sorption, saturating the samples; samples were marked with the letters ZX-WS-WV.

All sorption experiments were performed in three consecutive sorption-desorption cycles in order to assess the regeneration properties of the material. Due to technical limitations in the case of conducting the process with water vapor, water vapor was present only during the first sorption cycle. To enable the experiments to be performed in cycles, a pressure-swing process was applied using a vacuum of 10−2 Pa.

## 3. Results

### 3.1. Fly ash composition and analysis of synthesized materials

**Figure 3:**
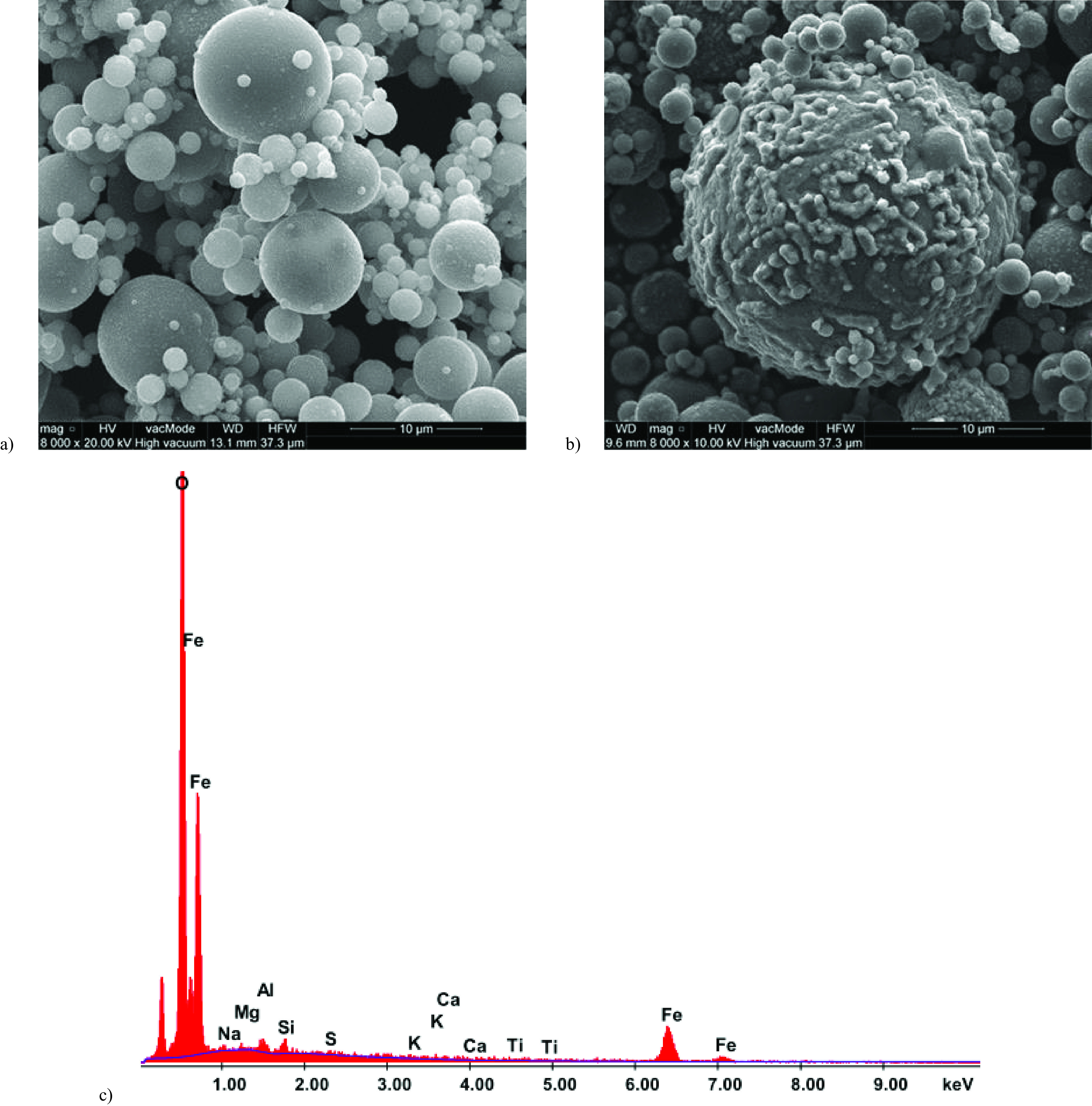
a) SEM micrograph of modified fly ash used for the synthesis and b) SEM micrograph of ferromagnetic residue from the demagnetization process; c) EDS analysis of the rough surface from the microphotograph (b).

The fly ash used for the synthesis is shown in Figure 3a, the residue from the magnetic separation process is shown in Figure 3b, and the EDS analysis of the rough surface visible in Figure 3b is presented in Figure 3c. The comparison shows that the process of magnetic separation successfully separated out the ferromagnetic components, which are characterized by a rougher surface [17,18]. The character of the separated structure was additionally confirmed by EDS analysis, which indicated a predominant share of Fe and O in the chemical composition.

For the experiment, modified fly ash with a chemical composition determined by the XRF method was used. The results of the analysis are presented in Table 1. Additionally, LOI analysis was performed on the LO fly ash according to the EN196-2:2005 standard. This categorization is based on the following assumptions: category A, ≤5.0% wt. LOI; category B, ≤7.0% wt. LOI; category C, ≤9.0% wt. LOI. The LOI value was 1.4% and this fly ash was accordingly classified as category A.

**Table 1:** Chemical composition of modified LO fly ash (main compounds).

LO fly ash	
Component	Content [%]
Na _2_ O	2.14
MgO	1.70
Al _2_ O _3_	15.44
SiO _2_	38.65
P _2_ O _5_	0.72
SO	1.35
K _2_ O	2.80
CaO	3.30
TiO _2_	0.99
Fe _2_ O _3_	7.10
ZnO	0.97

**Figure 4:**
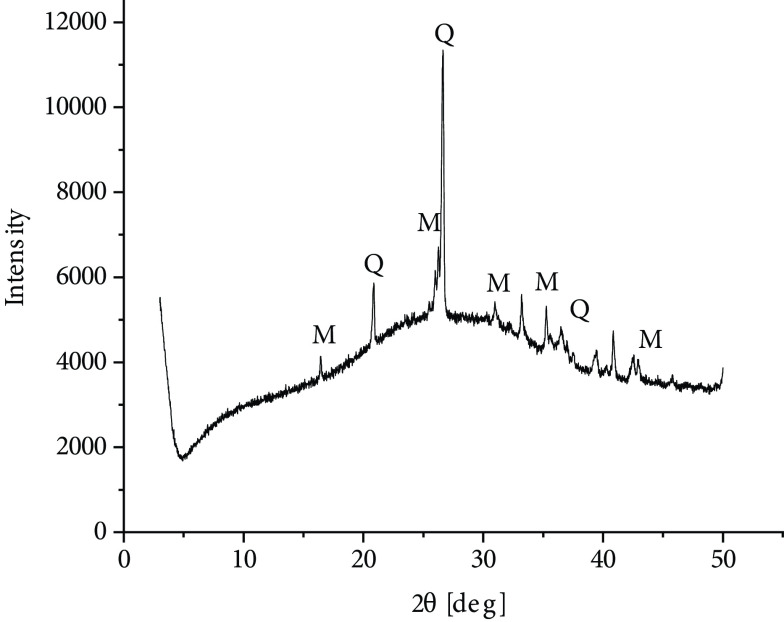
XRD diffractogram of modified LO fly ash: M – mullite, Q – quartz.

One of the additional parameters that can be given for zeolites is their Si/Al ratio [19]. For zeolite X in the literature, the Si/Al ratio is in the range of 1–1.5 [20]. Based on the XRF data obtained in this study, a value of 2.5 can be found for raw material fly ash. It should be noted, however, that in the case of fly ash zeolites, the remains of nonreacted fly ash are also present in the sample and therefore the Si/Al ratio may not give the correct value for the crystalline zeolite phase. Therefore, XRF analysis for fly ash zeolites was not performed. Fly ash used in the experiments was analyzed using the XRD method, as shown in Figure 4. Aluminosilicate glass was dominant in the mineral composition of the investigated fly ash, constituting 60% by mass. Crystalline phases were represented by mullite and smaller amounts of quartz, hematite, and magnetite.

**Figure 5:**
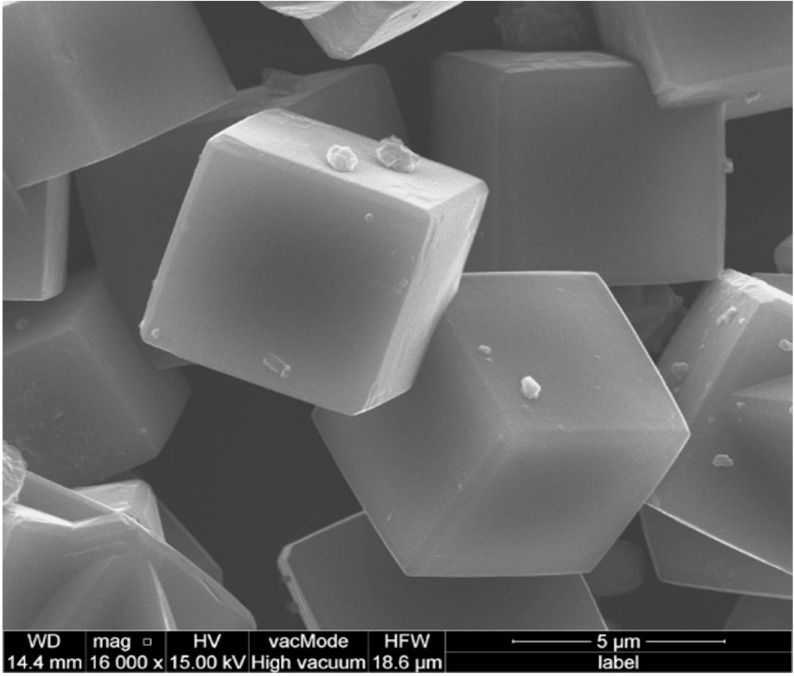
SEM micrographs of zeolite X.

The morphological forms of the obtained zeolites are presented in Figure 5. Na-X zeolite crystals are characteristic forms, with an isometric section and dimensions of 3–7 μm, often mutually overgrown.

**Figure 6:**
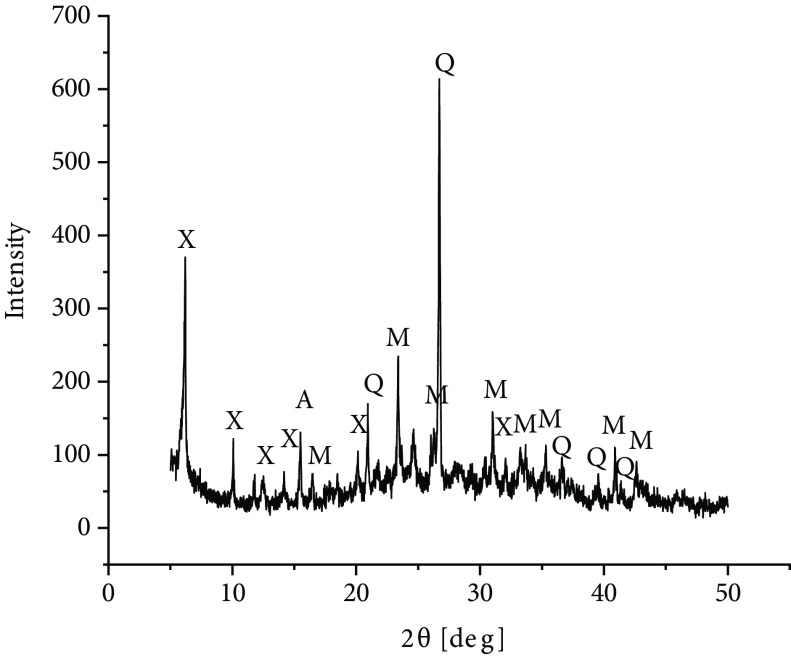
XRD diffractogram of material synthesized from LO fly ash for zeolite X: X – zeolite X, M – mullite, Q – quartz.

In order to confirm the presence of the desirable zeolite phase in the synthesized samples, XRD analysis was performed. Figure 6 shows a diffractogram of a sample of zeolite X synthesized from fly ash. Synthesis conditions were selected in such a way that only one zeolite phase was obtained in the sample. Zeolite X was recognized by the presence of the most intensive reflections in the diffractograms present at 2θ : 6.12◦ , 10◦ , 11.73◦ , 15.43◦ , 18.42◦ , 20.07◦ , 22.47◦ , and 23.31◦ .

Often during zeolite synthesis from fly ash, mixtures of different types of zeolites are obtained [4], which was undesirable in this investigation.

The amount of synthesized zeolite in the research material was 87% wt. Quantitative analysis of the mineral composition of zeolitic materials was based on Rietveld refinement. The remaining mineral components were mullite, quartz, and unreacted aluminosilicate glass residue.

### 3.2. SO _2_ sorption experiments on zeolite X

**Figure 7:**
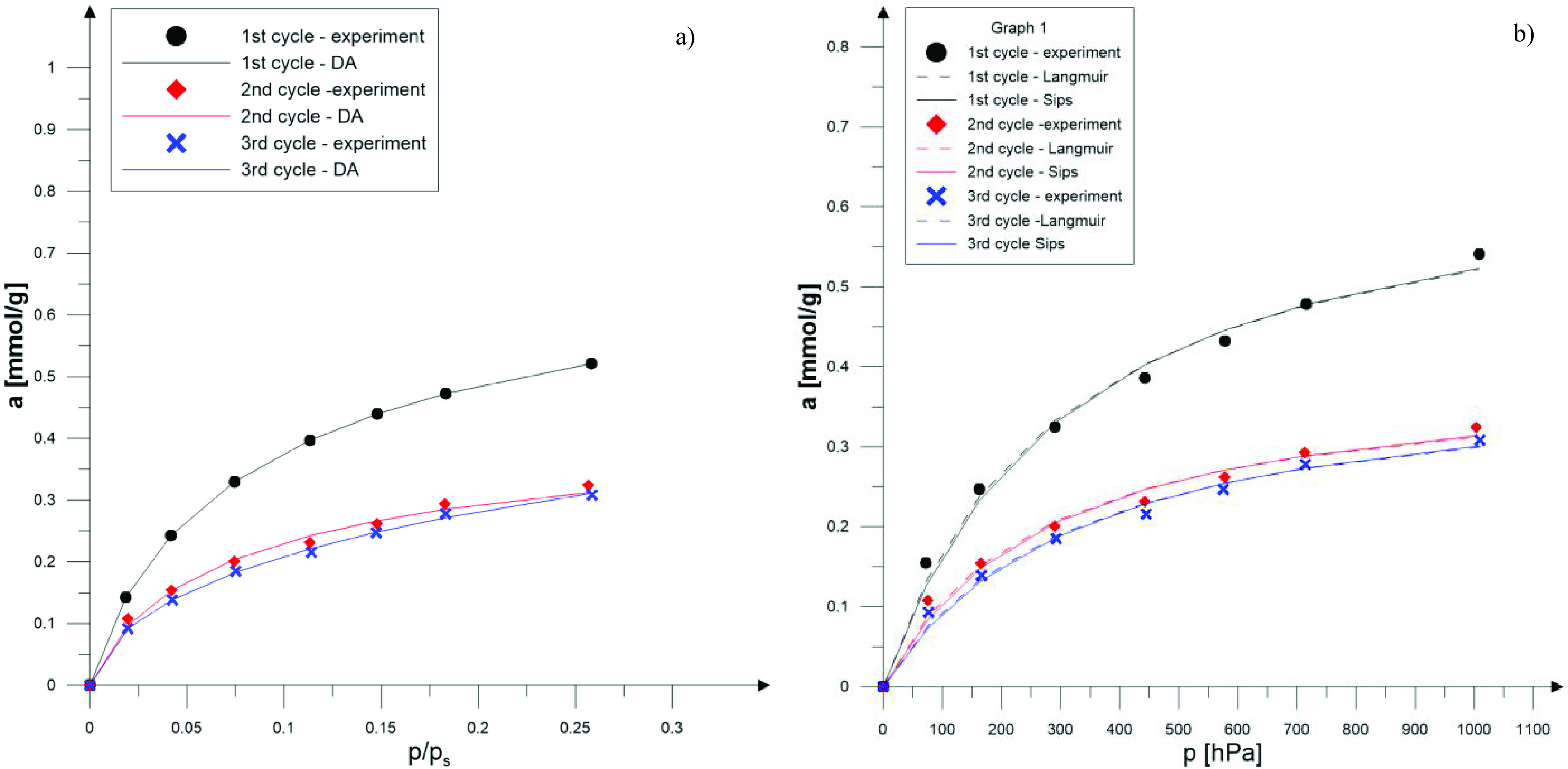
SO _2_ isotherm (T = 298 K) for ZX-WS for three sequential sorption-desorption cycles and its compatibility to DA (a) and Langmuir and Sips (b) models.

**Figure 8:**
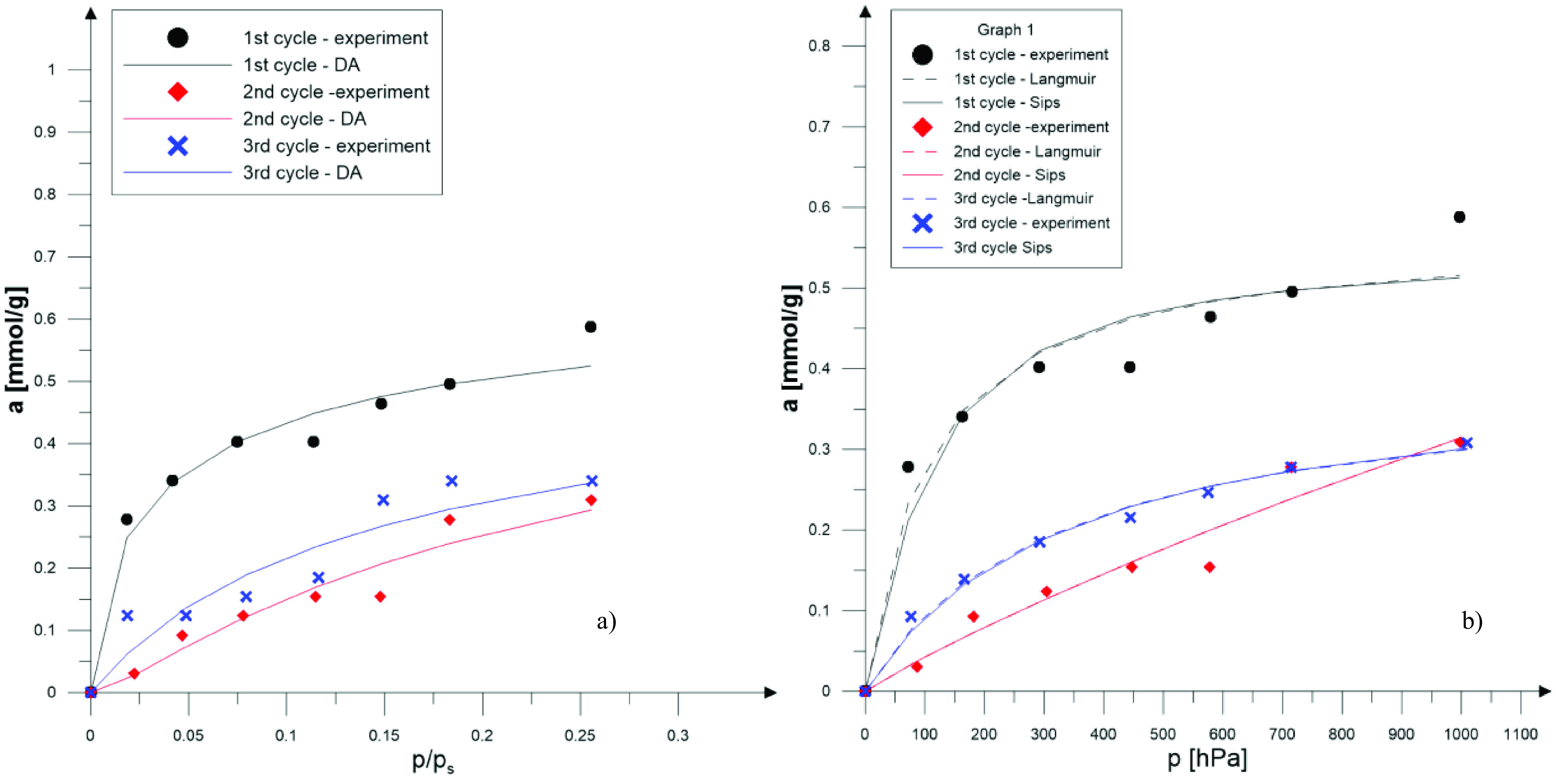
SO _2_ isotherm (T = 298 K) for ZX-DS for three sequential sorption-desorption cycles and its compatibility to DA (a) and Langmuir and Sips (b) models.

**Figure 9:**
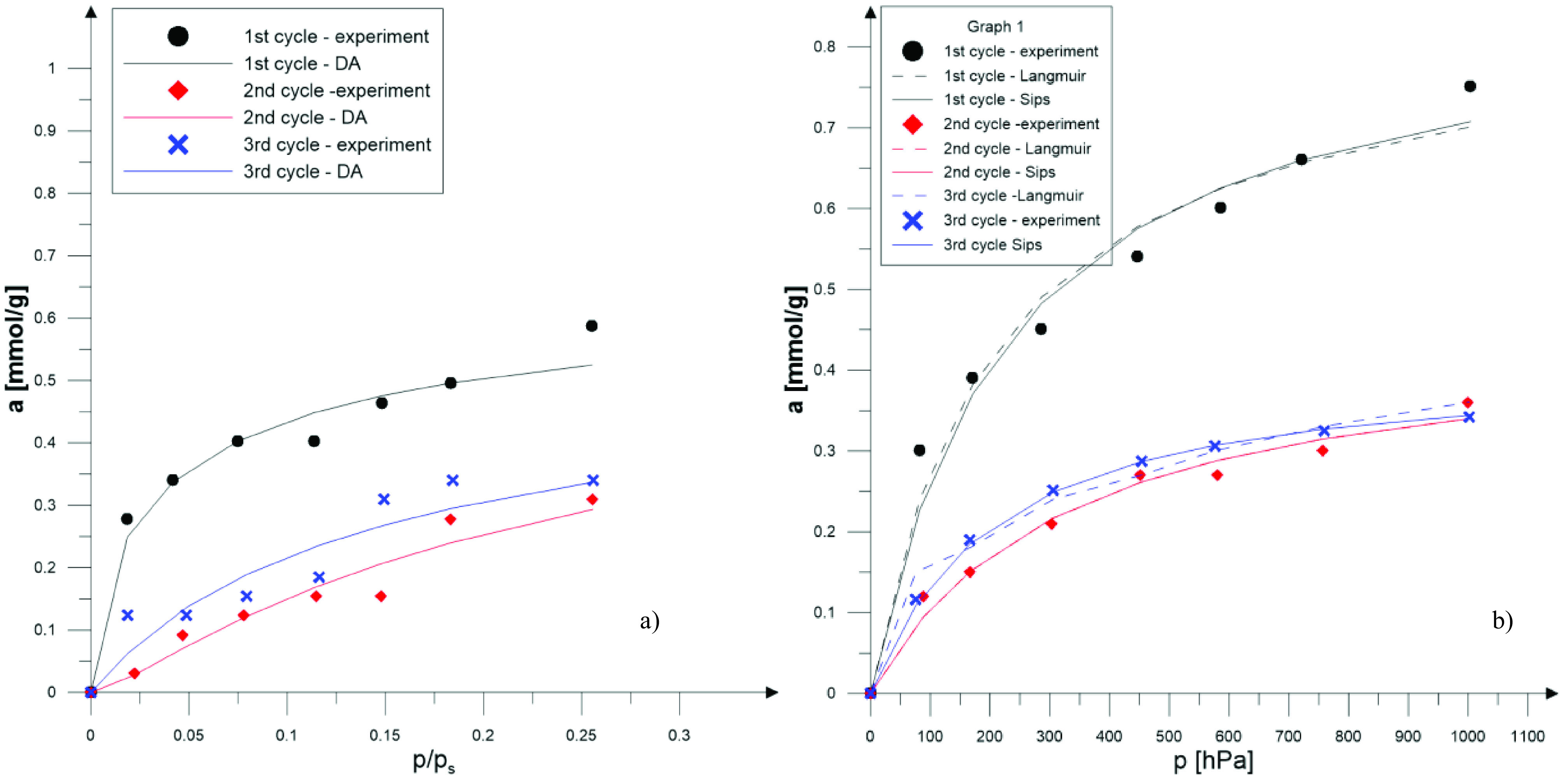
SO _2_ isotherm (T = 298 K) for ZX-WS-WV for three sequential sorption-desorption cycles and its compatibility to DA (a) and Langmuir and Sips (b) models.

The SO _2_ sorption experiments were performed on zeolite X samples obtained from fly ash. Three versions of each experiment were performed. Note that in the figures below (Figures 7a, 7b, 8a, 8b, 9a, and 9b), experimental data are shown along with theoretical isotherms calculated using the Dubinin–Astakhov (DA), Langmuir, and Sips theoretical models, as described in the following sections.

In Figure 7a and Figure 7b, SO _2_ adsorption isotherms for ZX-WS are shown.

In Figure 8a and 8b, SO _2_ adsorption isotherms performed on a ZX-DS sample are presented.

In Figure 9a and Figure 9b, SO _2_ adsorption isotherms performed on a ZX-WS-WV sample are presented. The sorption process was performed in the presence of water vapor.

Table 2 presents a comparison of the obtained SO _2_ sorption values. For all isotherms, it was found that the examined material had good regeneration properties. After the first cycle, a decrease in sorption capacity was observed, but in subsequent cycles, this remained at the same level.

**Table 2:** Comparison of SO _2_ sorption values for zeolite X.

	a [mmol/g]		
	1st cycle	2nd cycle	3rd cycle
ZX-WS	0.54	0.32	0.31
ZX-DS	0.59	0.31	0.34
ZX-WS-WV	0.75	0.36	0.36

### 3.3. Dubinin–Astakhov, Langmuir, and Sips models

The experimental results were described with the use of three adsorption isotherm models: Langmuir (Eq. 1), Sips (Eq. 2), and Dubinin–Astakhov (Eq. 3), in the following forms:

(1)a=am(L)KLP1+KLP(2)a=am(S)(KSP)1/n1+(KSP)1/n(3)V=V0exp[-(AβE0)n],A=-ΔG=RTlnp0p

Here:

K _L_ , K _S_ are Langmuir and Sips constants [h Pa ^-1^ ],

1/n is the Sips isotherm exponent,

V is the volume of adsorbate filling the micropores [cm ^3^ /g],

V _0_ is the total micropore volume [cm ^3^ /g],

p _s_ is the saturated vapor pressure [hPa],

E _0_ is the characteristic energy of adsorption for standard vapor [kJ/mol],

n is an equation parameter,

β is the affinity coefficient of the characteristic curves (for SO _2_ , β = 0.7),

A is the adsorption potential (the change of molar free energy related to the change of vapor pressure) [J/mol].

The Langmuir and Sips equations are the simplest adsorption equations. While the Langmuir equation assumes no interaction on the surface of the adsorbent and the homogeneous nature of the surface, the Sips equation allows heterogeneity of the adsorbent surface and is similar in its construction to the Freundlich adsorption isotherm. The DA equation is valid for the micropore filling mechanism over a range of p/p _0_ varying from 10 ^-7^ to 10 ^-2^ [21].

## 4. Discussion

All of the sorption tests were performed three times on the same sample. This was done in order to enable a preliminary check on whether the used material, irrespective of the preparation procedure, underwent a process of regeneration. It is worth noting that after the first cycle, the adsorption capacity decreased in all cases, but in the subsequent cycles, based on the available data, it seems that the sorption capacity remained relatively constant. The reason for this is probably the chemical reaction taking place during the first cycle between SO _2_ and the unwashed sodium hydroxide from the synthesis process.

It was assumed that chemisorption was possible in all cases. Sulfur dioxide and NaOH from the process of synthesis could react as follows (Eq. 4 and Eq. 5):

(4)SO2+NaOH< = >NaHSO3NaHSO3+NaOH< = >Na2SO3+H2O

The highest SO _2_ sorption capacity values in the first sorption-desorption cycle were found for the process of sorption in the presence of water vapor (sample ZX-WS-WV). In the literature sources, information on the positive influence of water vapor in the process of SO _2_ sorption can be found [14]. The chemical reaction between NaOH solution droplets and SO _2_ is exothermic and increases the temperature of the particle to the extent that water can be evaporated. The reaction proceeds by the formation of SO _2_ −3 initially and reverse hydrolysis to HSO−3 may occur if the particle does not crystallize because of excessive water evaporation. If the particle does crystallize, the lack of sufficient water for reverse hydrolysis leaves a Na2 SO crystalline microsphere as the reaction product [22]. Additionally, it was assumed that if no water was present, kinetic limitations could occur, as the anticipation time was the same for each isotherm point for all sorption experiments. For ZX-WS and ZX-DS, the values obtained for the first sorption-desorption cycle were slightly lower than in the case of sorption in the presence of water vapor. The chemisorptions with the remains of sodium hydroxide from the synthesis process took place only in the first sorption cycle. It was confirmed by the relatively constant sorption capacity value in the second and third cycles. In this case, however, the predominant mechanism was physical adsorption. In the case of additional drying of the samples, the sorption capacity was higher in comparison to the ZX-WS sample. This phenomenon is associated with the fact that the samples that were not carefully dried before the sorption most probably captured impurities from the air, which led to a decrease in the sorption capacity. In all sorption experiments, the sorption capacity after the second and third sorption cycles was on a very similar level. In this case, the preparation procedure, as well as the presence of water vapor, did not influence the sorption process, as all the samples were regenerated using low-pressure desorption. The design of the experiment was restricted by the technical limitations of the experimental set-up. The water vapor was introduced only in the first sorption cycle. The slightly higher values of sorption capacity in the case of the second and third sorption cycles of sample ZX-WS-WV were noted. It was assumed that the NaOH present in the pores after the process of synthesis was dissolved in water and partly removed in the process of desorption.

The authors wished to compare the sorption behavior of zeolite X synthesized from fly ash with other data in the literature; however, it was difficult for the following reasons. Very often, a mixture of zeolites is obtained in the process of synthesis from fly ash and the ratios of fly ash transformation into zeolites vary, which means that the amount of zeolite in the sample is variable. The authors therefore assumed that the most reasonable approach would be to compare the sorption capacities of pure Na-X zeolites in the 2.03–2.68 mmol/g range [23]. Taking into account the lower zeolite content in the sample and the preliminary check of its ability to regenerate, along with the usage of waste material as a substrate, it may be concluded that these materials can be used as SO _2_ sorbents.

In order to calculate the constants for the Langmuir and Sips equations, nonlinear fitting was used by applying the Solver tool.

In Table 3, a comparison of the Langmuir and Sips constants is presented.

**Table 3:** Langmuir and Sips constants obtained as a result of nonlinear approximations performed using the Solver tool.

Sample	Sorption cycle	a _m_ (L) [mmol/g]	K _L_ × 10 ^3^ [h Pa ^-1^ ]	a _m(S)_ [mmol/g]	K _S_ × 10 ^3^ [h Pa ^-1^ ]	n	R ^2^_(L)_	R ^2^_(S)_
ZX-WS	I	0.677	3.43	0.676	3.29	0.973	0.987	0.989
	II	0.394	3.82	0.396	3.68	0.977	0.992	0.993
	III	0.394	3.15	0.399	3.02	0.989	0.986	0.989
ZX-DS	I	0.569	9.65	0.548	9.43	0.836	0.887	0.888
	II	1.339	0.31	2.726	0.10	1.095	0.957	0.959
	III	0.624	1.34	2.697	0.07	1.427	0.851	0.853
ZX-WS-WV	I	0.846	4.82	0.863	4.42	0.983	0.975	0.977
	II	0.444	3.20	0.450	3.06	0.989	0.988	0.989
	III	0.406	5.33	0.407	5.07	0.952	0.978	0.979

As a result of the numerical calculations, the correlation coefficient was the lowest for ZX-DS. For other samples, the correlation coefficient was higher than 0.97. Based on the calculated constants for the Langmuir and Sips isotherms, the descriptions of the experimental results are given. Isotherms for all samples are shown in Figures 7b, 8b, and 9b.

Analyzing the obtained values of constants, especially for the monolayer sorption capacity, amL and amS values at an unrealistic level of several mmol SO _2_ per gram of zeolite were observed. This effect was caused by the experimental isotherm shape. Langmuir and Sips adsorption isotherms describe adsorption systems whose shapes are classified as type I in the IUPAC classification system. There were no significant differences in the quality of fit for the two isotherms. For systems in which all three cycles followed the type I shape, a double decrease in the am parameter was observed after the first adsorption cycle, with stabilization after the next adsorption cycle.

A formal description of the experimental data was given using the DA equation (Eq. 3). In this case, the constants of the equation were determined based on a nonlinear fitting using Solver. Very good and good fits were obtained for all examined systems. The calculated constants and correlation coefficients are compared in Table 4.

**Table 4:** Results of fitting of DA isotherm to sulfur dioxide isotherms for fly ash zeolite X.

Sample	Sorption cycle	V _0_ [cm ^3^ /g]	E _0_ [kJ/mol]	n	R ^2^
ZX-WS	I	0.029	11.60	1.69	0.993
	II	0.017	12.01	1.95	0.987
	III	0.017	11.46	1.95	0.990
ZX-DS	I	0.027	15.32	1.93	0.889
	II	0.020	7.89	1.88	0.913
	III	0.021	9.81	1.85	0.821
ZX-WS-WV	I	0.038	12.81	1.93	0.965
	II	0.019	11.50	1.95	0.970
	III	0.019	13.13	1.94	0.973

Based on the determined constants, isotherms of the adsorbed quantity of SO _2_ as a function of relative pressure were drawn (Figures 7a, 8a, and 9a).

The volume of micropores V0 is a measure of the SO _2_ sorption limit for the sorption system. These values show that the maximum sorption limit with respect to SO _2_ was highest for zeolite X saturated with water vapor. For all cases, a twofold and even sometimes threefold reduction of the exhaust gas V0 parameter was observed after the first cycle, as well as a slight change in this parameter in the next cycle.

## 5. Conclusions

As a result of this research, it was found that zeolite X synthesized from fly ash is a good SO _2_ sorbent. The presence of water vapor in the process of sorption leads to a higher sorption capacity. In the case of ZX-WS and ZX-DS, the sorption capacity was higher for the additionally dried sample, as a process of vacuum drying allowed for the desorption of all impurities present in the pores of the sorbent. Regeneration tests provided confirmation that the samples presented regeneration properties. After the first cycle, the sorption capacity was reduced, which is probably related to the phenomenon of irreversible chemisorption on the remains of the hydroxide solution from the synthesis process. For sample ZX-WS-WV, it was assumed that the dissolution of sodium hydroxide and its removal in the desorption process took place.

Based on the calculated fittings, it can be stated that the DA equation best describes the systems studied. The Langmuir and Sips adsorption isotherms give similar accuracy with respect to the experimental data, but the results are not credible, since the shape of the experimental isotherm does not follow that of type I experimental isotherms according to the IUPAC classification system.
